# A practical introduction to sequentially Markovian coalescent methods for estimating demographic history from genomic data

**DOI:** 10.1002/ece3.5888

**Published:** 2019-12-07

**Authors:** Niklas Mather, Samuel M. Traves, Simon Y. W. Ho

**Affiliations:** ^1^ School of Life and Environmental Sciences University of Sydney Sydney NSW Australia

**Keywords:** coalescent, demographic history, mutation rate, population genomics, population size

## Abstract

A common goal of population genomics and molecular ecology is to reconstruct the demographic history of a species of interest. A pair of powerful tools based on the sequentially Markovian coalescent have been developed to infer past population sizes using genome sequences. These methods are most useful when sequences are available for only a limited number of genomes and when the aim is to study ancient demographic events. The results of these analyses can be difficult to interpret accurately, because doing so requires some understanding of their theoretical basis and of their sensitivity to confounding factors. In this practical review, we explain some of the key concepts underpinning the pairwise and multiple sequentially Markovian coalescent methods (PSMC and MSMC, respectively). We relate these concepts to the use and interpretation of these methods, and we explain how the choice of different parameter values by the user can affect the accuracy and precision of the inferences. Based on our survey of 100 PSMC studies and 30 MSMC studies, we describe how the two methods are used in practice. Readers of this article will become familiar with the principles, practice, and interpretation of the sequentially Markovian coalescent for inferring demographic history.

## Graphical abstract

A common goal of population genomics and molecular ecology is to reconstruct the demographic history of a species of interest. In this practical review, we explain some of the key concepts underpinning sequentially Markovian coalescent methods. Readers of this article will become familiar with the principles, practice, and interpretation of these methods for inferring demographic history.

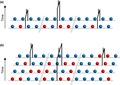

## 1 INTRODUCTION

The genomes of organisms carry a record of the evolutionary and ecological forces that have shaped their populations. In principle, one can reconstruct the demographic history of a species from the genome sequences of its present‐day representatives (Beichman, Huerta‐Sanchez, & Lohmueller, 2018). These reconstructions can be used to answer various biological questions, such as the influence of climatic events on population size and structure (Miller et al., 2012), the timing of major events in the evolutionary history of modern humans (Fu et al., 2014; Li & Durbin, 2011), human impacts on wild animal populations (Johnson et al., 2018; Pujolar, Dalén, Hansen, & Madsen, 2017), and the effects of domestication (Frantz et al., 2016; Yu et al., 2018).

Demographic inference is rarely straightforward; there are many challenges in extracting the relevant historical signal from the population of interest. A number of methods have been developed for this purpose, such as those based on site frequency spectra and approximate Bayesian computation (reviewed by Beichman et al., 2018). Here, we focus on a pair of methods designed to analyze genome sequences from small samples of individuals: the pairwise sequentially Markovian coalescent (PSMC; Li & Durbin, 2011) and multiple sequentially Markovian coalescent (MSMC; Schiffels & Durbin, 2014). We focus on these methods because they are widely used, they share a common theoretical framework, and a large amount of work has been done on elucidating their strengths and weaknesses. Even as new methods of demographic inference are published, interpreting the large number of studies that have already used PSMC and MSMC is difficult without a solid understanding of the assumptions and approximations made by their underlying models.

The PSMC method can be used to analyze unphased sequence data from a single diploid individual, whereas MSMC can use sequences from several individuals. The two methods are particularly useful when studying deeper population timescales and when there are very limited numbers of samples (Beichman et al., 2018; Spence, Steinrücken, Terhorst, & Song, 2018). For example, PSMC has been employed to great effect in studies of individual ancient samples, including those of an ancient horse (Orlando et al., 2013), an ancient wolf (Skoglund, Ersmark, Palkopoulou, & Dalén, 2015), and two woolly mammoths (Palkopoulou et al., 2015).

In addition to reconstructing demographic history, PSMC and MSMC have been used to infer the timing of population divergence and to estimate mutation rates from ancient genomes. However, various studies have demonstrated that inferences from the two methods can be sensitive to violations of the underlying assumptions (e.g., Mazet, Rodríguez, Grusea, Boitard, & Chikhi, 2016), and the methods do not offer an explicit framework for testing hypotheses. As a consequence, the user needs some appreciation of the underlying biological theory and statistical methods to ensure that the results are interpreted appropriately. In this review, we give a brief description of coalescent theory, the mathematical framework behind demographic inference, before discussing how it is applied in PSMC and MSMC. We describe some of the key practical issues that arise when these two methods are applied to genomic data.

### 1.1 Coalescent theory

Coalescent theory provides a statistical framework that relates the size of a population to the coalescence times in the genealogy of the sampled individuals (Hudson, 1983; Kingman, 1982; Tajima, 1983). Coalescent models envision the evolutionary process running backwards in time: We start from the leaves of the genealogy, which represent the individuals that have been sampled in the data set. We then trace out their full evolutionary history by following their lineages back in time. Coalescence events occur whenever two lineages combine to become one ancestral lineage. If the population is panmictic (random mating), then all possible pairs of lineages have an equal probability of coalescing.

The rate of coalescence can tell us about population size because coalescence events are more likely to occur when the population is small. For example, if we select a few people at random from a small, isolated village, they are likely to share an ancestor in recent generations. If a few people are chosen at random from the entire human population, they are unlikely to be closely related; we would probably need to look much further into the past before we would find a coalescence event.

Coalescent theory makes this idea mathematically rigorous by providing formulae that relate the rate of coalescence to the effective population size. Thus, if we have a genealogy that shows when the coalescent events occurred, we can infer how the size of the population changed over time (Pybus, Rambaut, & Harvey, 2000). Time periods with many coalescence events indicate a smaller population, and vice versa. A number of tools have been developed for inferring demographic history from the coalescence times in individual gene trees; these methods have been reviewed elsewhere (Ho & Shapiro, 2011).

The coalescent framework usually involves the assumption of neutral evolution. However, various forms of selection act across genomes, and these can affect specific mutations as well as any neutral variants that are linked (Maynard Smith & Haigh, 1974). Estimates of population size can be distorted by natural selection (Ewing & Jensen, 2016; Schrider, Shanku, & Kern, 2016). In particular, purifying selection is likely to be predominant and tends to remove genetic variation, which would lead to an apparent reduction in population size (Charlesworth, 1994; Charlesworth, Morgan, & Charlesworth, 1993). These potential impacts need to be borne in mind when using any methods of inference based on the coalescent, especially if the data set includes large sections of coding sequence.

### 1.2 The sequentially Markovian coalescent

How can PSMC use coalescent theory to infer demographic history from a single genome, given that we need data from multiple coalescence events that each requires two alleles? The answer is that each genome contains large numbers of loci, which can split apart from each other during recombination and so trace out distinct evolutionary histories. By tracking the coalescence events between the two alleles at every locus, we can infer how many of them have occurred across the genome within a given time interval. PSMC and MSMC use this information to reconstruct the effective population size through time, provided that we make some assumptions about the mutation rate.

The original coalescent theory did not provide a framework for efficiently estimating population sizes from individual genomes, so it required modification before it could be used to infer population‐size history using the approach described above. Before explaining the theoretical advances that solved this problem, we should consider what sort of model would be useful for inference. Obviously, the model should be biologically reasonable. It should also be mathematically tractable, in the sense that the computation of the likelihood functions for parameters is feasible. One class of models that fit this criterion, and hence are used widely in biology, are hidden Markov models (Box 1; Rabiner, 1989; Zucchini, MacDonald, & Langrock, 2016). In this framework, the data that we see are generated by a hidden background process. The process switches between a number of states, each of which has a specified probability of producing each of the observations. We cannot know with certainty what the background process is doing when we observe the data, but the observations provide probabilistic information about the process. Additionally, the background process must be Markovian, which means that the next state of the process depends only on the current state.

Box 1 **Hidden Markov models**

A hidden Markov model is a pair of stochastic processes, $X_{t}$ and $Y_{t}$, where $X_{t}$ is the “hidden process” and cannot be directly observed, but $Y_{t}$ can. At each point *t,* $X_{t}$ takes on one of N possible states according to some specified probability distribution. Because $X_{t}$ is a Markov process, the state it takes on depends only on the state at $X_{t-1}$. After $X_{t}$ has moved to its new state, the value of $Y_{t}$ is generated by a probability distribution that depends on the value that $X_{t}$ takes on at that time. The values that $Y_{t}$ can take are typically referred to as the “observation symbols” of the process.

To create a hidden Markov model, we need to define the key ingredients of the process that we described above:

1. The possible states of $X_{t}$, $q_{i}$, i∈{0, …, *N*}
2. The possible observation symbols $v_{i},\;i\in \left\{0,\ldots ,M\right\}$
3. A probability distribution, the “transition probabilities,” that describes how we move between the states of $X_{t}$: $P(X_{t+1}=q_{j}|X_{t}=q_{i}$).
4. A set of probability distributions, called the “emission probabilities,” that describe how the states of $X_{t}$ generate the values of $Y_{t}$. Each of these will be of the form $b_{j}\left(k\right)=P(Y_{t}=v_{k}|X_{t}=q_{j})$.
5. A probability distribution describing how the system looked when *t* = 1: $P(q_{i}\left|t=1\right)$.

In the case of the sequentially Markovian coalescent models, *t* indexes locations along the genome. The hidden states are characterized by the local genealogies at each locus. For PSMC, the possible states are the possible coalescence times of the two alleles. For MSMC, it is the coalescence time of the two alleles in the sample that coalesce first. The observation symbols are features of the genetic data. For PSMC, the data are partitioned into bins of 100 bp; we observe a 1 if a heterozygous locus occurred in that bin and a 0 otherwise. For MSMC, there are a few more observation symbols to account for the extra complexity introduced by multiple genomes. The emission probabilities are determined by the mutation rate and the transition probabilities by the recombination rate.

Much of the power of hidden Markov models for inference is that an algorithm—the Baum–Welch algorithm—exists that allows us to compute estimates of all of the free parameters simultaneously, provided that we specify the underlying structure of the process and how the process relates to the observations (Zucchini et al., 2016). Thus, the problem of inference under the coalescent could be solved by finding a biologically accurate description of the coalescent with recombination as a hidden Markov model. This was achieved by a change in perspective. Instead of starting from extant samples and building the full genealogy by working backwards, we work our way along the genome (Wiuf & Hein, 1999). We start from one end of the genome and then generate a “local” genealogy for each locus by incorporating new information as we move along the genome and encounter recombination events (Marjoram & Wall, 2006; McVean & Cardin, 2005; Wiuf & Hein, 1999). In the language of hidden Markov models, the local genealogy is the background process that generates the data, whereas the sequences are the observations. This process was named the sequentially Markovian coalescent (McVean & Cardin, 2005).

**Figure 1:**
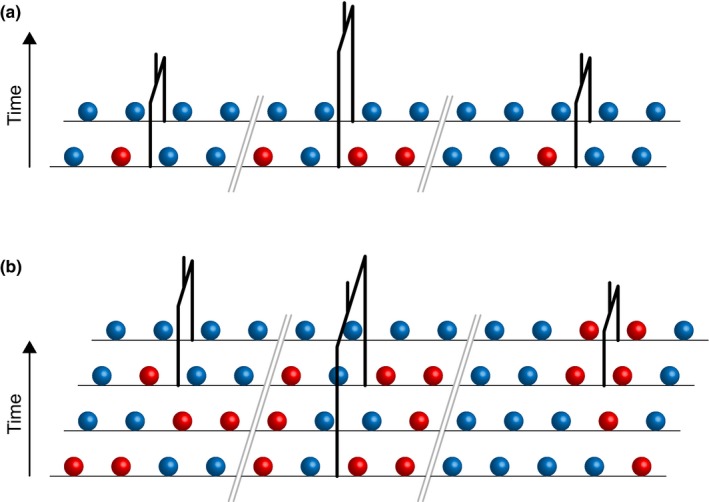
The sequentially Markovian coalescent. The colored circles represent nucleotide states belonging to the alleles at each locus. Double gray lines denote recombination breakpoints, which separate the loci along the genome. The time to the most recent common ancestor of the two alleles at each locus is reflected in the local tree. (a) In PSMC, there are only two haplotypes. Thus, the topology of the local tree is fixed, but the time to the most recent common ancestor differs among loci. (b) In MSMC, there are multiple haplotypes. MSMC ignores most of the local tree topology, focusing only on the most recent coalescence event at each locus

In PSMC, the local genealogy is completely characterized by the time to the most recent common ancestor of the two alleles, because there is only one possible tree topology for two leaves (Figure 1a). Analyzing multiple genomes is much more computationally challenging, but the MSMC simplifies this task by using only a subset of the local tree that describes the time to the most recent common ancestor of the two alleles that coalesce first at that locus (Figure 1b). The complexities associated with the analysis of multiple genomes have been addressed differently in other coalescent hidden Markov models (Dutheil, 2017).

## 2 APPLICATIONS OF THE METHODS

### 2.1 Reconstructing population sizes

The PSMC and MSMC methods were originally designed to infer past changes in effective population sizes, including the timing of expansions and bottlenecks. The output of each method is a plot showing how the effective size of the population to which that individual belonged has changed over time. PSMC and MSMC can combine information from much larger numbers of loci than is computationally feasible with tree‐based methods such as skyline plots. For this reason, sequentially Markovian coalescent methods can probe deeper timescales, because the data are much more likely to include loci that have their most recent common ancestor far into the past. For example, application of the PSMC has been able to shed light on more than a million years of the demographic histories of modern humans and other great apes (Prado‐Martinez et al., 2013). Although neither PSMC nor MSMC provides a formal framework for testing specific hypotheses of the causes of population‐size changes, bootstrap replicates can be used to approximate a confidence interval around the estimate of effective population size (Li & Durbin, 2011).

In principle, MSMC has superseded PSMC, even when only a single diploid genome is available for analysis. MSMC estimates the recombination rate correctly in circumstances where PSMC fails to do so (Schiffels & Durbin, 2014). When multiple genomes are available, MSMC has better power to resolve recent changes in effective population size, because adding alleles increases the chance that there will be a coalescence event in the recent past. In analyses of human genomes, MSMC is informative for events that occurred as recently as 2,000 years ago (Schiffels & Durbin, 2014), whereas PSMC does not reliably resolve changes in effective population size that had occurred within the last 20,000 years (Li & Durbin, 2011).

There are limits to how far in the past the sequentially Markovian coalescent can make reliable estimates of demography. For the models to infer the population size in a given time period, the genome must have loci at which alleles coalesced in that period. However, coalescent theory shows that alleles with deep coalescences are relatively rare (Takahata & Nei, 1985). As we look further back in the past, it becomes increasingly unlikely that we have any data on the coalescence rate. Consequently, the inference of coalescence rates, and hence population sizes, becomes much noisier for the deep demographic past.

A critical consideration when using the PSMC and MSMC is that their output cannot always reliably be interpreted as plots of population‐size changes. Recall from our description of the model that it estimates the rate of coalescence at each point in time. Coalescent theory shows that the inverse of this rate, sometimes called the inverse instantaneous coalescence rate (Mazet et al., 2016), can be used as a proxy for population size under certain assumptions. However, if the study population does not meet these assumptions, then other factors can affect the coalescence rate and must be taken into account when we attempt to interpret apparent changes in population size (Chikhi et al., 2018; Mazet, Rodríguez, & Chikhi, 2015; Mazet et al., 2016). For example, the relationship between coalescence times and population sizes can be confounded by natural selection and by nonrandom mating (Mazet et al., 2016).

One intuitive example of nonrandom mating is the *n*‐island model, where *n* panmictic populations are separated except for some fixed rate of migration (Wright, 1931). Because two alleles cannot coalesce while they are in different islands, the expected coalescence time is determined by the number of islands and the migration rate between them, as well as by the population size (Mazet et al., 2016; Pannell, 2003). In particular, when the migration rate between islands is low, the coalescent effective population size—the parameter inferred by sequential methods—can be much greater than the true population size (Li & Durbin, 2011; Nei & Takahata, 1993). Thus, peaks on the demographic plot might correspond to periods of increased population structure rather than increased population size.

The assumption of panmixia can also be violated by inbreeding, which increases the rate of coalescence and hence lowers the effective population size. It should manifest in the genome as long runs of homozygous sequence (Ceballos, Joshi, Clark, Ramsay, & Wilson, 2018). One strategy for testing whether inbreeding has affected demographic inference is to identify and remove such runs of homozygosity, then repeating the analysis and checking for any changes in the inferences (Freedman et al., 2014).

In general, there is great difficulty in differentiating between the changes that are attributable to shifts in population size and those that are caused by changes to other demographic parameters (such as increased migration or a strengthening of population structure). Changes in some demographic parameters can alter the demographic curve in ways that are not well localized to the point at which the changes occurred. For example, because the migration rate is changed instantaneously in a constant‐sized *n*‐island population, the demographic curve rises and falls over many thousands of generations (Mazet et al., 2016). When many changes happen over a short space of time, they can interact in complex ways to produce the demographic plot, so it is difficult to attribute any feature of the plot to a specific change.

We do not know exactly what degree and type of structure will preclude the reliable interpretation of the inferences from PSMC and MSMC, but their use for highly structured populations is likely to be extremely misleading. Unfortunately, the impact of population structure cannot be assessed directly from the observed coalescence times. Even when the population size is constant but the coalescence time varies purely as a result of changes in population structure, we can always find a set of (false) population‐size changes that would explain the observed coalescence times arbitrarily well (Mazet et al., 2016). Additionally, testing for population structure typically requires data from many individuals, which might not be available when methods based on the sequentially Markovian coalescent are used.

Finally, there are reasons to believe that the sequentially Markovian coalescent might perform poorly on realistic data. For example, when genetic data are produced by simulation under demographic models inferred by MSMC from human genomes, they fail to resemble the empirical data in important ways (Beichman, Phung, & Lohmueller, 2017). Other methods that use data from many individuals, such as δaδi (Gutenkunst, Hernandez, Williamson, & Bustamante, 2009) and SMC++ (Terhorst, Kamm, & Song, 2017), perform substantially better in this regard. Concerningly, this problem appears to grow worse as larger numbers of genomes are used for the MSMC, so the problem cannot necessarily be overcome by adding more data.

Since the release of MSMC, new methods have been designed that expand on the framework provided by the sequentially Markovian coalescent (Dutheil, 2017; Spence et al., 2018). These methods can outperform PSMC and MSMC in certain ways. The SMC++ method allows much larger numbers of genomes to be used for inference than is computationally possible with MSMC and does not require the genomes to be phased (Terhorst et al., 2017). SMC++ is more accurate than MSMC, especially for population sizes in the recent past and when phasing error is present. Another method, MAGIC (minimal‐assumption inference from population‐genomic data; Weissman & Hallatschek, 2017), is conceptually similar to the sequentially Markovian coalescent in that it infers the coalescent history of a sample from the distribution of polymorphisms in the genome. However, it does not use an explicit model of coalescence and recombination and can estimate many different parameters from the empirical data. Given the different strengths and weaknesses of the various methods for inferring demographic history, best practice should include the use of multiple methods and comparison of their inferences (Spence et al., 2018).

### 2.2 Studying changes in population structure

A common task in population genetics is to infer the timing of divergence between closely related populations or species. One method that can be used for this purpose is the multispecies coalescent (e.g., Ogilvie, Bouckaert, & Drummond, 2017). However, this method requires data from multiple individuals per species and is not computationally feasible for data sets comprising large numbers of loci. Both PSMC and MSMC can be used to infer divergence times while incorporating information from whole genomes.

**Figure 2:**
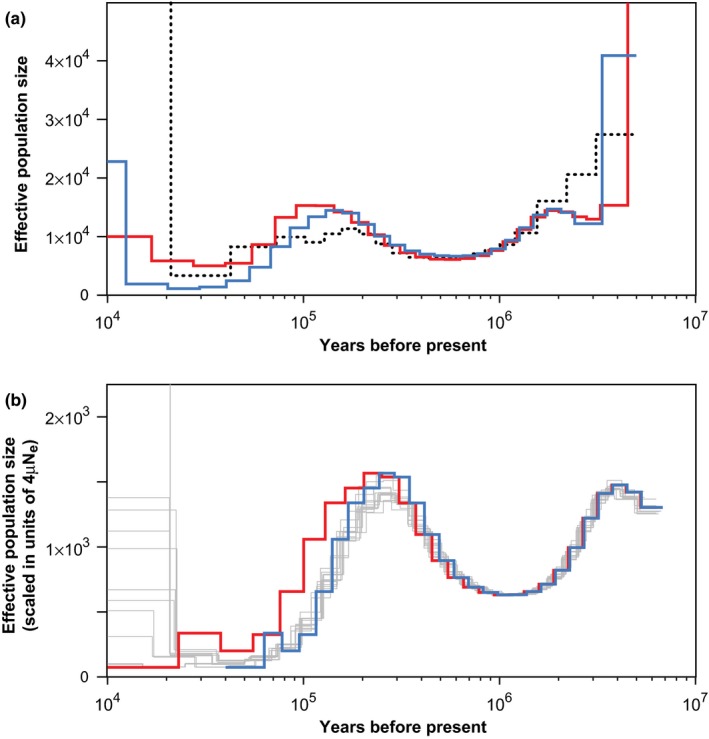
Illustrations of two different uses of PSMC and MSMC methods. (a) Dating speciation events using the PSMC (Li & Durbin, 2011). The solid lines represent PSMC plots from the genomes of two modern humans: Yoruba (red) and Chinese (blue). Looking backwards in time, the plots become identical from about 100–120 thousand years ago, indicating a shared population history. The dotted line shows a PSMC plot from a hybrid genome constructed from the X chromosomes of the Yoruba and Chinese genomes, scaled by 0.75. Looking forwards in time, the infinite population size at about 20 thousand years ago suggests cessation of gene flow between the two populations. (b) Estimating the mutation rate using PSMC (Fu et al., 2014). The two thick lines are plots for a 45,000‐year‐old modern human from Ust'‐Ishim in western Siberia, either uncorrected (red) or shifted horizontally (blue) to align with the plots from present‐day non‐African modern humans (thin gray lines). The magnitude of the horizontal shift indicates the number of mutations that have occurred in 45,000 years, providing a means of estimating the mutation rate

Although it is not an intended use of the method, PSMC can be adapted to identify the point at which gene flow ceased between a pair of populations (Cahill, Soares, Green, & Shapiro, 2016; Li & Durbin, 2011). A simple approach is to compare PSMC plots obtained from representatives of the two populations or species. The point at which their plots become identical indicates when they represent the same ancestral population (Figure 2a).

An alternative PSMC‐based approach uses a synthetic diploid genome that is constructed from phased or unphased haplotypes from the two populations or species. The synthetic genome is intended to mimic that of an F_1_ hybrid and so the approach is known as hPSMC (Cahill et al., 2016). Coalescence between the two alleles at each locus can only occur in the ancestral population. Because the rate of coalescence drops to zero after the populations have become reproductively isolated from each other, the effective population size will then be inferred to be infinite (Figure 2a). This technique can only provide a maximum bound on the divergence time, because it is possible that the populations diverged later than the most recent coalescence event. On the other hand, the estimated timing of divergence can be misled if the populations have not achieved complete reproductive isolation. Given the sensitivity of hPSMC to any gene flow that has occurred after the divergence between the two populations, the method is more appropriately used to date the cessation of gene flow rather than population divergence (Cahill et al., 2016).

Multiple sequentially Markovian coalescent tends to perform better across a range of population divergence scenarios (Zhou & Teo, 2016) and can be used to provide a more complete picture of how gene flow between populations has changed through time. If we know which samples came from which populations, MSMC can calculate the cross‐coalescence rate between each pair of populations, as well as the coalescence rate within each population (e.g., Fan et al., 2019; Liang et al., 2019). The ratio of these terms will grow or shrink depending on the amount of gene flow between subpopulations: When migration between populations is high for some period of time, the cross‐coalescence rate between populations should increase gradually during that interval as the populations come to share more alleles (Schiffels & Durbin, 2014).

### 2.3 Estimating mutation rate

The PSMC framework has been used to estimate mutation rates from genome sequences (e.g., Fu et al., 2014; Palkopoulou et al., 2015; Skoglund et al., 2015). The technique requires two sequences that have distinct ages and that have been sampled from the same population. Given their shared demographic history, the two genomes should yield very similar plots of effective population size. However, the two plots will be offset along the time axis because of the difference in sampling times (Figure 2b). The mutation rate can be estimated by finding the number of mutations that need to be added to the ancient sequences in order to superimpose the two plots, then dividing this by the difference in the ages of the two samples. One can do this numerically by simply adding mutations to the older genome until its demographic plot coincides with that of the younger genome, but the estimation can be performed more rigorously by using a maximum likelihood approach (Fu et al., 2014).

Inferring mutation rates using PSMC plots should only be done when an estimate of the mutation rate for the target species is otherwise unavailable. The use of demographic plots for this purpose is subject to a host of confounding factors, including those that affect the standard use of PSMC to infer population‐size history. Other more generally accepted methods for estimating mutation rates, particularly those based on genome sequences from parent‐offspring sets (e.g., Besenbacher, Hvilsom, Marques‐Bonet, Mailund, & Schierup, 2019; Roach et al., 2010) or mutation‐accumulation lines (e.g., Ossowski et al., 2010), are likely to be substantially more accurate.

## 3 DATA REQUIREMENTS

### 3.1 Genome sequences

Both PSMC and MSMC require data in the form of one or more genome sequences aligned to a reference sequence from the target species or from a closely related species. Using a reference sequence that is too dissimilar can reduce the accuracy of heterozygote calls (Günther & Nettelblad, 2019). Prior to analysis, genome data should be filtered to remove sites that have a high probability of being called inaccurately (Li, 2014). Failing to filter appropriately, especially when coverage is low, can lead to the demographic signal being obscured (Nadachowska‐Brzyska, Burri, Smeds, & Ellegren, 2016).

The PSMC method requires a diploid consensus genome and does not require that haplotypes are phased, because it only needs to know the nucleotide positions that are heterozygous. The genome must be sequenced to a sufficiently high degree of coverage that heterozygous sites can be called accurately; one estimate is that 18‐fold coverage and <25% missing data are required for reliable inference (Nadachowska‐Brzyska et al., 2016). PSMC might still be able to recover the essential features of a demographic history at lower coverage, but the shape of the graph tends to be flattened so that estimates of the effective population size might not be accurate. The effect of low coverage is worse when the amount of data is small, as when the analysis is based on only a subset of the genome.

Whole genome sequences should be used in principle, but informative PSMC plots can be obtained from individual chromosomes from the human genome (Li & Durbin, 2011). A recent simulation study also showed that PSMC can obtain accurate estimates even when using the products of short‐read sequencing assembled into short scaffolds (Gower et al., 2018). If a subset of the genome is chosen, coding DNA should not constitute the majority of the data set because of the confounding effects of selection on demographic inference. Excluding sequences that are close to coding regions or that have low recombination rates can also help to mitigate the confounding impacts of selection (Schrider et al., 2016).

The MSMC method was designed to use data from multiple haplotypes but can use as few as two, in which case it reduces to a variant of PSMC (referred to as PSMC'). There is no upper bound on the number of haplotypes that can be used, but the method is feasible for at least eight haplotypes and the computational complexity increases rapidly as more are added (Schiffels & Durbin, 2014). When there are too many haplotypes to be used efficiently in MSMC, other methods can handle data sets comprising larger numbers of genomes (e.g., SMC++ and MAGIC; Terhorst et al., 2017; Weissman & Hallatschek, 2017).

In principle, MSMC requires that the sequence data are phased (i.e., haplotypes are specified), unless working with data from a parent–parent–offspring trio or from a single diploid genome. However, the importance of the phasing depends on the question that one seeks to answer: MSMC performs reasonably well when inferring the shape of a demographic curve from unphased data, but there is a substantial reduction in its resolution of the recent past and its ability to infer population divergence times (Schiffels & Durbin, 2014).

Where phasing is performed, it needs to be highly accurate because even a relatively small rate of phasing error can seriously mislead the effective population sizes estimated by MSMC, especially for the recent past (Song, Sliwerska, Emery, & Kidd, 2017; Terhorst et al., 2017). This can be problematic for MSMC, because phasing to the required level of accuracy will typically need a larger sample size or access to an external reference panel of haplotype information (Browning & Browning, 2011). When robust phasing is not possible, using unphased data might be a better option if one is only interested in the qualitative shape of the demographic curve. Alternatively, one can use a phasing‐invariant method of demographic inference, such as SMC++, if the goal is to obtain precise estimates of population size or to explore demographic events from the recent past (Browning & Browning, 2011; Terhorst et al., 2017).

### 3.2 Restriction‐site‐associated DNA data

Both PSMC and MSMC can be used with restriction site‐associated DNA (RAD) data (Liu & Hansen, 2017). RAD sequencing is a reduced‐representation method that gives the sequences of regions flanking the cutting sites of a chosen restriction enzyme (Miller, Dunham, Amores, Cresko, & Johnson, 2007). The smaller the fraction of the genome that this subset covers, the greater the reduction in accuracy and increase in variance. As with inferences from other reduced data sets, the demographic curve obtained from RAD data is flatter, with peaks and troughs that are less pronounced (Liu & Hansen, 2017).

Based on evidence from simulations, a rule of thumb is that PSMC can recover the broad shape of the demographic curve if *μp*/*r* > 0.5, where *μ* is the mutation rate, *p* is the fraction of the genome covered by the RAD sequencing, and *r* is the recombination rate (Liu & Hansen, 2017). In practice, when RAD data are used for demographic inference, the read length and sampling density should be maximized.

## 4 PRACTICAL CONSIDERATIONS

### 4.1 Parameter selection

In PSMC and MSMC analyses, a number of settings need to be specified by the user. The two methods assume that the history of the population is divided into discrete time intervals on which the population size is constant. The user must specify the length and number of these intervals; a poor choice of intervals can lead to over‐ or underfitting of the model. Repeating the analysis using different numbers of time intervals can show whether there is any impact on the inferences (Nadachowska‐Brzyska et al., 2016).

Intuitively, splitting a time period into many intervals can lead to greater stochastic error in the population‐size estimate for each time interval, because there will be higher variance in the number of coalescent events occurring in smaller intervals. In addition, because coalescent events are rarer when the population is large, model overfit will be most pronounced at the “peaks” of the plot. One way to reduce the impact of this problem is to check that a sufficiently large number of coalescent events fall into each interval. This threshold is somewhat subjective, but a minimum of 20 events per interval has been suggested (Li & Durbin, 2011). To choose an appropriate number of time intervals, it might be useful to begin with some number of evenly spaced intervals. After running the analysis, one can identify sections of the plot with too few coalescent events and reduce the number of intervals in those periods before repeating the analysis.

The above argument also shows a weakness of the PSMC and MSMC methods. We cannot distinguish between the noise induced by overfitting and the signals of genuine changes in population size. Thus, one should be very cautious in interpreting changes in effective population size on small timescales, especially around peaks of the demographic plot. Creating bootstrap replicates of the analysis can provide an estimate of the variance in the estimated effective population size (Li & Durbin, 2011).

Both PSMC and MSMC also require the user to supply an initial value for the ratio of the mutation rate and recombination rate. In theory, this should not affect the outcome of the analysis because it only provides a starting value for the algorithm. Simulations have shown that even when the analysis fails to estimate this value, there are no negative impacts on the estimates of population sizes (Li & Durbin, 2011).

### 4.2 Scaling the graphs

Analyses using PSMC and MSMC give plots of effective population sizes that are scaled to the per‐generation mutation rate. To allow a time axis to be added to the plot, the mutation rate (per generation) and generation interval need to be specified. Generation interval can be difficult to define precisely, even for well‐studied taxa such as modern humans (Scally & Durbin, 2012). Reliable estimates of mutation rates are not easily obtained, because phylogenetic estimates of long‐term evolutionary rates are not necessarily applicable at the population level (Ho, Duchêne, Molak, & Shapiro, 2015); rates of spontaneous mutation have been inferred for a limited number of eukaryote species (Besenbacher et al., 2019; Smeds, Qvarnström, & Ellegren, 2016). If estimates of the mutation rate are unavailable for the target species, common practice has been to employ the phylogenetically closest estimate (see Section 5). An alternative approach is to derive an approximation of the mutation rate based on its covariation with other biological quantities, such as genome size and population size (Lynch et al., 2016). This method has been used in a number of PSMC and MSMC studies (e.g., Hall et al., 2017).

Using an incorrect value for either the mutation rate or generation interval does not affect the qualitative shape of the plot, and so will not affect analyses that do not require the precise dating of demographic events. A higher mutation rate will cause the estimated population size to be scaled down linearly and will shift the curve closer to the present, whereas a longer generation interval will scale the population size down (Nadachowska‐Brzyska et al., 2016). If precise dating is required, it might be best to run the analyses under a plausible range of mutation rates, to provide upper and lower bounds for the dating of demographic events.

## 5 USAGE SURVEY

To present a picture of how PSMC and MSMC are used in scientific studies, we surveyed their usage in peer‐reviewed journal articles. We randomly sampled 100 of the ~200 studies that have performed PSMC analysis and 30 of the ~60 studies that have performed MSMC analysis. We identified these studies by scanning the approximately 1,400 papers that have cited the original descriptions of the two methods (Li & Durbin, 2011; Schiffels & Durbin, 2014), according to Google Scholar.

**Figure 3:**
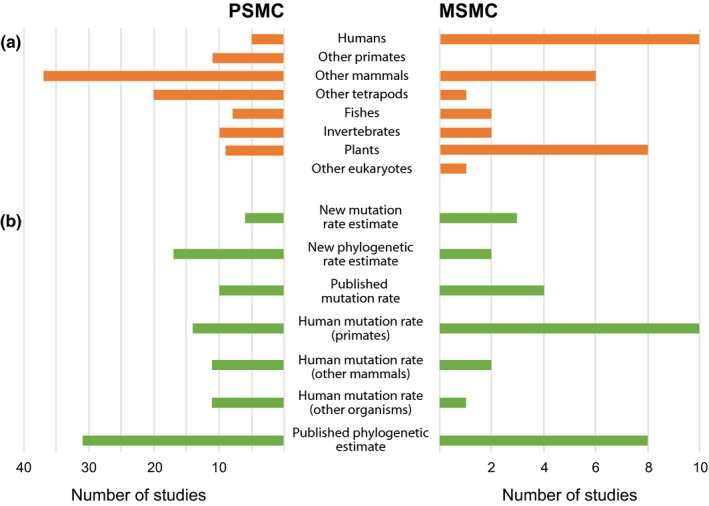
Data from random samples of 100 studies that implemented the pairwise sequentially Markovian coalescent (PSMC) method and 30 studies that implemented the multiple sequentially Markovian coalescent (MSMC) method. (a) Taxonomic affiliations of the organisms studied. (b) Source of mutation rates used to scale the demographic plots. The choice of mutation rate affects the scale of the horizontal axis and the estimate of the effective population size, but does not affect the qualitative shape of the plot. For example, a higher mutation rate will cause the estimated population size to be scaled down linearly and will shift the curve closer to the present. Details of the 130 studies surveyed are provided in Appendix S1 on Dryad (https://doi.org/10.5061/dryad.0vt4b8gv2)

Among the 100 PSMC studies that we examined in detail (Figure 3a), the majority focused on the genomes of mammals (53%) and other vertebrates (28%). Although many of these studies investigated population structure and estimated rates of gene flow between subpopulations, these demographic features were very rarely taken into account during interpretation of the PSMC plots. Similarly, the potential confounding impacts of selection were sometimes acknowledged, but were not addressed explicitly. Among the 30 MSMC studies that we examined in detail, there tended to be a greater focus on modern humans and plants (Figure 3a).

The vast majority of the PSMC and MSMC studies analyzed whole genome sequences, although some produced separate demographic plots for autosomes and sex chromosomes (e.g., Ekblom et al., 2018; Foote et al., 2016). Most studies did not provide explicit justification for the choice of discrete time intervals, which was usually the default number of 64 based on the initial application of PSMC to human genomes (Li & Durbin, 2011). Among the 30 MSMC studies, 11 analyzed data sets with two haplotypes (equivalent to the PSMC) and 11 analyzed data sets with eight haplotypes.

The PSMC and MSMC plots were usually scaled according to mutation rates that had been estimated in previous studies (Figure 3b). Many of these mutation rates were estimated on phylogenetic scales, but they were more often obtained from pedigree‐based analyses. Estimates of human mutation rates were applied to PSMC and MSMC plots in 49 studies, although only 24 of these involved analyses of humans or other primates. Studies of birds, flies, lepidopterans, and plants tended to apply mutation rates estimated from *Ficedula* (Nadachowska‐Brzyska et al., 2016), *Drosophila* (Haag‐Liautard et al., 2007), *Heliconius* (Keightley et al., 2015), and *Arabidopsis* (Ossowski et al., 2010), respectively; together these accounted for 11% of the studies surveyed. Even in these cases, however, the scaling of the demographic plots can be severely misled if there is substantial rate heterogeneity among species. Only 7% of studies produced novel estimates of short‐term mutation rates for the target species, for example using analyses of parent–parent–offspring trios (Künstner et al., 2016; Martin et al., 2018). Our usage survey highlights the challenges in identifying suitable estimates of mutation rates for rescaling the demographic plots produced by PSMC and MSMC.

## 6 CONCLUSIONS

Sequentially Markovian coalescent methods provide powerful means of inferring demographic histories from genomic data. They are particularly useful when the genome sequences are restricted to only a few individuals, or when the aim is to probe timescales that are inaccessible to other methods (Beichman et al., 2018). However, there are substantial challenges in interpreting the output of PSMC and MSMC, because testing the assumptions of the underlying models usually requires more data than are available when these methods are used. Nevertheless, by being aware of the underlying theoretical framework and identifying the assumptions made in the interpretation of the results, researchers can glean valuable demographic information that might otherwise be unavailable.

## CONFLICT OF INTEREST

None declared.

## AUTHOR CONTRIBUTIONS

S.M.T. and S.Y.W.H. performed the usage survey. N.M. and S.Y.W.H. wrote the paper, with input from S.M.T.

## Data Availability

Data collected for Figure 3 are available provided in Appendix S1 on Dryad (https://doi.org/10.5061/dryad.0vt4b8gv2).
